# Methadone, Metoclopramide and Metronidazole Interaction Causing Torsades de Pointes

**DOI:** 10.3390/clinpract11010015

**Published:** 2021-02-07

**Authors:** Karthik Gnanapandithan, Nishrutha Karthik, Jaime Gerber

**Affiliations:** 1 Department of Internal Medicine, Mayo Clinic, 3500 San Pablo Road S, Jacksonville, FL 32224, USA; 2 Department of Internal Medicine, Yale University School of Medicine, New Haven, CT 06510, USA; Nithik23@gmail.com; 3 Department of Cardiology, Yale University School of Medicine, New Haven, CT 06510, USA; Jaime.Gerber@yale.edu

**Keywords:** QT prolongation, Torsades de Pointes, metoclopramide, metronidazole, methadone, drug-induced Torsades de Pointes

## Abstract

There are several classes of medications that can cause prolongation of the corrected QT (QTc) interval and potentially Torsades de Pointes (TdP). Most of these medications are commonly used in the emergency department, and interaction between these medications increases the risk of this iatrogenic complication. We describe a patient on methadone therapy who developed TdP after she received metoclopramide and metronidazole. Interaction between different classes of medications can increase the risk of QTc prolongation and TdP. Awareness of this condition and its risk factors need continuous reinforcement among all hospital personnel to reduce the risk of this life-threatening complication.

## 1. Introduction

There are multiple factors that can affect myocardial repolarization and result in prolongation of the corrected QT (QTc) interval [1,2]. Abnormal prolongation of the QTc interval can result in Torsades de Pointes (TdP), which is a type of polymorphic ventricular tachyarrhythmia. Most of the medications that were initially described to affect the QT interval were antiarrhythmics; however, a large number of non-cardiac drugs are now known to be associated with this adverse event. In recent times, drug-induced long QT syndrome (LQTS) is the most important cause of the withdrawal of marketed drugs [1]. The interaction can occur due to multiple drugs that prolong the QT interval or a medication that potentiates the effect of another by affecting its metabolism. We describe a case in which interaction between multiple medications, namely methadone, metoclopramide and metronidazole, resulted in TdP.

## 2. Case Report

**Figure 1:**
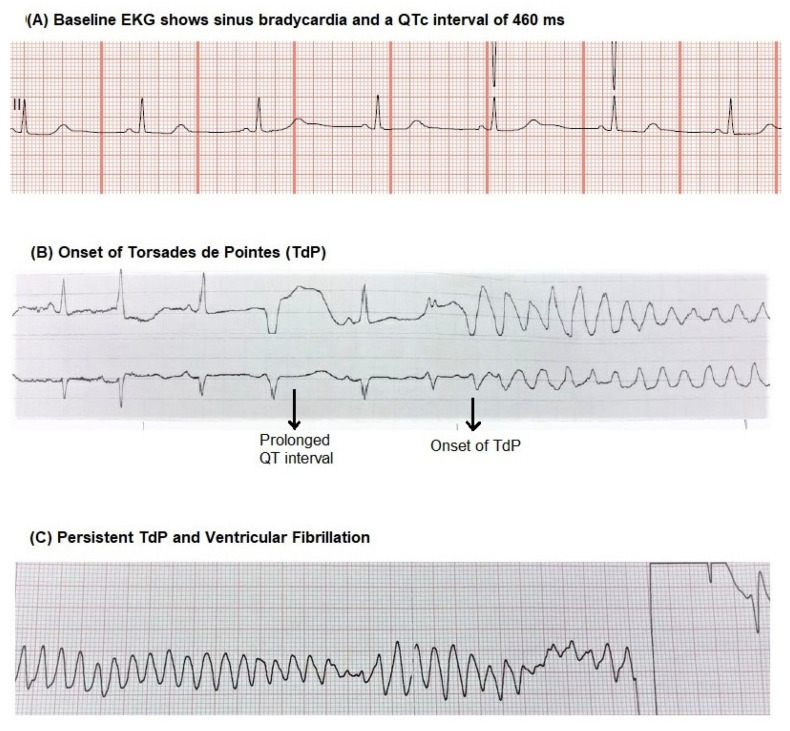
(**A**) Electrocardiogram on admission shows prolonged corrected QT (QTc) and variability in QT interval. (**B**) Onset of Torsades de Pointes (TdP) shortly after metronidazole infusion. (**C**) Persistent TdP requiring defibrillation.

A 50-year-old female with a medical history of acquired immunodeficiency syndrome not compliant with anti-retroviral therapy, polysubstance abuse and opioid dependence on methadone maintenance was brought in to the emergency department (ED) after she was found vomiting and confused in the streets. She reported using heroin and cocaine. On evaluation, she had sinus bradycardia, with a heart rate of 44 per minute, respiratory rate of 18 per minute and blood pressure of 128/80 mm Hg. Examination revealed a lethargic patient with no focal neurological deficits, clear lungs, sinus rhythm with no murmurs and soft abdomen. Laboratory testing showed a serum potassium of 3.5 meq/L, magnesium 2.1 meq/L, creatinine 0.5 mg/dL. Electrocardiogram (EKG) revealed sinus bradycardia with a rate of 40, a QT interval of 512 ms and a corrected QT (QTc) of 460 ms (Figure 1A). A non-contrast computerized tomography of the head and a chest radiograph did not show any acute abnormality. Her urine toxicology was positive for methadone, cocaine and opioids. Her mental status slowly improved during her stay in the ED; however, she started to have nausea and vomiting. She received intravenous (IV) metoclopramide 10 mg. She developed a fever of 101F during her stay in the ED. She was given IV ceftriaxone and metronidazole for suspected aspiration pneumonia. Metronidazole was started about 45 min after the metoclopramide was given and was slowly infused over 30 min. Shortly after the metronidazole infusion was stopped, she was unresponsive and the cardiac monitor (Figure 1B,C) revealed Torsades de Pointes (TdP). She was defibrillated with a return to sinus rhythm. Follow up EKG showed sinus rhythm with a QTc of 542 ms. Cardiology was consulted. IV potassium chloride and magnesium sulfate were given. Later, an echocardiogram showed an ejection fraction of 47%, normal right ventricular function and no valvular abnormalities. Over the next two days, there were no significant arrhythmias and the QTc returned to 432 ms. 

## 3. Discussion

Abnormal repolarization pattern resulting from prolongation of QT interval or abnormal morphology of the T-wave can result in TdP [1,2]. Congenital and acquired causes of long QT syndrome (LQTS) can predispose to TdP. Apart from a prolonged QT, the presence of bradycardia and beat-to-beat variability [3,4] in QT are other known risk factors for TdP. Medications that affect cardiac repolarization are a common cause of acquired LQTS. In the acquired variant, a typical sequence of events has been described in the EKG [2]. It starts with a ventricular premature complex or an ectopic beat that results in a compensatory pause. This is followed by a sinus beat that usually has a long QTc, terminating in a ventricular extrasystole that usually starts the TdP. It may be self-limiting, in which case it can cause syncope, or if sustained, it can degenerate into ventricular fibrillation resulting in a cardiac arrest. The EKG strip in our patient (Figure 1A) shows the variability in QT interval and prolongation of QT before the onset of TdP. 

Drug-induced LQTS that can potentially lead to TdP and ventricular fibrillation arrest is a well-known but underestimated problem. Antiarrhythmics, psychiatric medications, antihistaminics, antibiotics, antifungals and antiemetics are some of the common classes of drugs that can cause prolongation of QTc. All these drugs are commonly used in the emergency department and inpatient setting [5]. There are reports of metoclopramide associated TdP [6,7], mostly in patients with underlying heart disease or renal failure. Cocaine [8,9] and methadone [10] are also associated with LQTS. Other cardiac effects of methadone predisposing to TdP include bradycardia and QT dispersion [11]. Opioids vary in their tendency to predispose to cardiac arrhythmias. Methadone has been identified as a higher risk agent that can increase the risk of long QT related arrhythmias even in lower doses [12], and hence, a close monitoring of these individuals is recommended. Methadone is metabolized in the liver, primarily by CYP3A4, with CYP 2D6 and CYP1A2 also being involved [13]. Metronidazole is an inhibitor of CYP2C9 and CYP3A4 isoenzymes and can interact with drugs [14] causing LQTS and TdP. Older age, female sex, underlying structural heart disease, recent myocardial infarction and family history of sudden cardiac death are known risk factors [1,15]. Simultaneous use of drugs that can prolong QT interval is a significant risk factor. Use of these drugs in patients on enzyme (cytochrome P450) inhibitors or those with electrolyte abnormalities (hypokalemia, hypocalcemia and hypomagnesemia) can also predispose to TdP. Judicious use of these drugs in recommended doses, minimizing use in patients with pre-existing prolonged QT interval or other cardiac risk factors can help in reducing this life-threatening iatrogenic adverse event. There are several online tools such as RxList, WebMD and Medscape that are available to health care professionals to check interactions between medications. CredibleMeds [16] is a website with a free smartphone app that offers information on general drug interactions and a dedicated section on QTc interactions. 

TdP with hemodynamic instability is treated with defibrillation. IV magnesium sulfate is the first-line therapy [17,18] and is effective in patients with normal serum magnesium levels as well. Other interventions include identification and discontinuation of the culprit medications and correction of electrolyte abnormalities, especially hypokalemia [18]. Cardiac pacing and isoproterenol are reserved for refractory cases [17,18].

## 4. Conclusions

Though well known, drug interactions leading to LQTS and TdP remain a dangerous yet underestimated problem. In patients on methadone or those with a prolonged QTc, caution should be exercised in the concurrent use of other medications that can interact to increase the risk of TdP. High-risk patients need to be on close cardiac monitoring when these drugs are being administered. Continued reinforcement of this condition among all hospital personnel can increase awareness and help reduce the incidence of this life-threatening complication.
